# Functional dissection of translocon proteins of the *Salmonella *Pathogenicity Island 2-encoded type III secretion system

**DOI:** 10.1186/1471-2180-10-104

**Published:** 2010-04-08

**Authors:** Stefanie U Hölzer, Michael Hensel

**Affiliations:** 1 Mikrobiologisches Institut, Universitätsklinikum Erlangen, Erlangen, Germany

## Abstract

**Background:**

Type III secretion systems (T3SS) are essential virulence factors of most Gram-negative bacterial pathogens. T3SS deliver effector proteins directly into the cytoplasm of eukaryotic target cells and for this function, the insertion of a subset of T3SS proteins into the target cell membrane is important. These proteins form hetero-oligomeric pores acting as translocon for the delivery of effector proteins. *Salmonella enterica *is a facultative intracellular pathogen that uses the *Salmonella *Pathogenicity Island 2 (SPI2)-encoded T3SS to manipulate host cells in order to survive and proliferate within the *Salmonella*-containing vacuole of host cells. Previous work showed that SPI2-encoded SseB, SseC and SseD act to form the translocon of the SPI2-T3SS.

**Results:**

Here we investigated the structural requirements of SseB and SseD to form a functional translocon. Based on bioinformatic predictions, deletional analyses of SseB and SseD were performed and the effect on secretion by the T3SS, formation of a translocon, translocation of effector proteins and intracellular replication was investigated. Our data showed that both SseB and SseD are very sensitive towards alterations of the primary structure of the proteins. Although proteins encoded by mutant alleles were still secreted, we observed that all mutations resulted in a loss of function of the SPI2-T3SS.

**Conclusion:**

These observations indicate that translocon proteins of the SPI2-T3SS are highly evolved towards the formation of multi-subunit complex in the host cell membrane. Structural alterations are not tolerated and abrogate translocon function.

## Background

Gram-negative bacteria have evolved various mechanisms for the transport of proteins across the bacterial envelope. Among these, type III secretion systems (T3SS) and type IV secretion systems are of specific interest since these systems mediate the vectorial transport of effector proteins into eukaryotic target cells [reviewed in [1]]. This process is termed translocation and requires the contact of the bacteria to a host cell membrane. T3SS are involved in a variety of bacteria-host cell interactions, ranging from symbiosis to pathogenesis [2]. Pathogenic bacteria deploy T3SS to translocate effector proteins with toxin-like activities and can manipulate various host cell functions by means of these effectors. *Salmonella enterica *is a facultative intracellular pathogen that has developed a unique intracellular lifestyle. *Salmonella *uses two distinct T3SS during different phases of pathogenesis [3]. The *Salmonella *Pathogenicity Island 1 (SPI1)-encoded T3SS mediates invasion of non-phagocytic cells and triggers inflammatory responses [reviewed in [3]].

During the intracellular phase of pathogenesis, *Salmonella *resides within a specific organelle of the host cell, the so-called *Salmonella*-containing vacuole or SCV. The biogenesis of the SCV and the intracellular survival and replication critically depend on the function of virulence genes clustered within *Salmonella *Pathogenicity Island 2 (SPI2), a locus that encodes a second T3SS [4]. The expression of SPI2-T3SS genes is induced in intracellular *Salmonella *and expression is controlled by the SsrAB two-component system. So far, the factors sensed by this system are not known.

Translocation by the T3SS requires the contact to a membrane of the host cell. On the molecular level, it has been demonstrated that the contact actually results in insertion of a subset of T3SS proteins into the target cell membrane [5]. These proteins are secreted substrate proteins of the T3SS but do not enter the host cytoplasm but rather form a complex in the target cell membrane. The hetero-oligomeric complex leads to the formation of a pore or translocon through which effector proteins enter the target cell. The analyses of various T3SS indicated that translocons are commonly composed of three subunits belonging to protein super-families [reviewed in [6]]. SPI2-encoded proteins are most similar to the T3SS proteins of enteropathogenic *E. coli *(EPEC) and a close evolutionary relationship between the systems has been proposed. EPEC translocon proteins are termed Esp. The EspA family of proteins is involved in the formation of a filamentous structure linking the T3SS in the bacterial envelope to the translocon pore in the target membrane. The EspD family consists of highly hydrophobic proteins which are membrane integral with several transmembrane helixes. EspB is a further protein required for translocation and with its homologs considered to be part of the translocation pore [6].

Previous molecular and functional characterization has revealed that SseB (EspA family), SseC (EspD family) and SseD (EspB family) are secreted substrate proteins of the SPI2-T3SS and required for the translocation of effector proteins by intracellular *Salmonella *[7]. We could also demonstrate that SseB, SseC and SseD are not required for formation of needle-like appendages on *Salmonella *cells, but are involved in the translocon formation in infected host cells [8].

While the structure-function relationship of translocon subunits has been analyzed in greater detail for the T3SS of EPEC, *Shigella *spp. and *Yersinia *spp., only little is known about the translocon subunits of the SPI2-T3SS. In this work, we performed a functional dissection of SseB and SseD, two subunits of the translocon of the SPI2-T3SS. This analysis indicates that SseB and SseD are highly sensitive towards alteration of the primary structure. All predicted domains in SseB or SseD are required for the function as translocon subunit, while secretion by the SPI2-T3SS can still take place after deletion of various protein domains.

## Results

### Deletional analyses of translocon proteins SseB and SseD

Based on the previous observation that SseB, SseC and SseD are required for the translocation of effector proteins by intracellular *Salmonella *[7], we started deletional analyses for the identification of functionally essential domains of the proteins. Here we focused on SseB and SseD.

Since SseB and SseD are most likely membrane-associated or integral proteins with hydrophobic character, the analysis of the hydrophobicity was a main consideration for the positions of deletions. In addition, coiled-coil domains are commonly found in substrate proteins of T3SS and have been shown as required for protein-protein interactions. The location of predicted coiled-coil domains in the sequence of SseB and SseD was also considered for the design of mutations.

**Figure 1:**
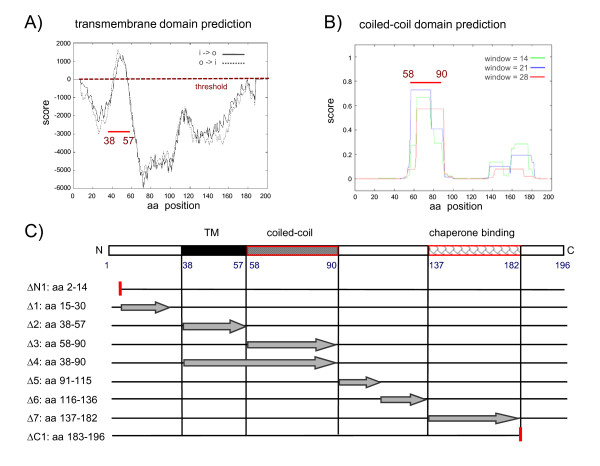
**Bioinformatic analyses of SPI2 translocon protein SseB and characteristics of deletion variants of SseB**. A) Using the program TMpred, putative transmembrane (TM) domains of the translocon protein SseB was predicted. o-i indicate the strongly preferred model, with N-terminus outside (aa 38-57), i-o indicates the alternative model. B) Using the program COILS, coiled-coil regions in SseB were predicted. As output option the default parameters were selected that gave residue number, residue type and the frame and coiled-coil forming probability obtained in scanning windows of 14, 21 and 28 residues (as described on the Swiss EMBnet homepage). The region spanning aa 58-90 was considered as coiled-coil domain. C) Schematic representation of the amino acid sequence of wild-type SseB and positions of deletions analyzed in this study. The predicted TM domain, coiled-coil region, as well as the chaperone-binding site [10] are indicated. The deleted regions within *sseB *variants are indicated by arrows and C- or N-terminal truncations are indicated by vertical red lines.

The hydropathy plots, predictions of coiled-coil domains and the positions of deletions are displayed in Fig. 1A. Briefly, SseBΔN1 lacked the N-terminal aa residues 2-14 and SseBΔ1 the N-terminal residues 15-30. SseBΔ2 was deleted for a hydrophobic region predicted as transmembrane region (aa 38-57), SseBΔ3 lacked the region containing coiled-coil domains (aa 58-90) and SseBΔ4 lacked both regions (aa 38-90). Constructs SseBΔ5 and SseBΔ6 were deleted for aa 91-115 or aa 116-136, respectively, both regions were without specific functional or structural predictions. SseΔ7 was deleted for the putative chaperone binding site, i.e. aa 137-182. Finally, SseBΔC1 was deleted for the C-terminal region of aa 183-196.

**Figure 2:**
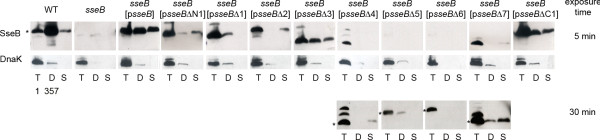
**Effect of various deletions in *sseB *on synthesis and secretion of SseB *in vitro***. *S*. Typhimurium WT or Δ*sseB *without plasmid, harboring plasmid p*sseB *for complementation of the *sseB *deletion, or plasmids for the expression of various *sseB *mutant alleles (p*sseB*Δx) were grown in 400 ml minimal medium PCN-P (0.4 mM) at pH 5.8 to induce SPI2 expression as well as protein secretion by the SPI2-T3SS. For analyses of protein synthesis, equal amounts of bacterial cells as adjusted by OD_600 _were harvested and resuspended in SDS-PAGE sample buffer (T, total cell fraction). Secreted protein bound to the bacterial surface was released by mechanical shearing and precipitated from bacteria-free supernatant (D, detached fraction) and secreted proteins in the supernatant were precipitated by addition of 10% TCA (final concentration). For Western blot analysis, samples corresponding to equal amount of bacteria or supernatant were separated by SDS-PAGE and transferred to nitrocellulose membranes and protein was detected with antiserum raised against SseB. As loading control and control for cell lysis, the bacterial heat shock protein DnaK was detected. The position of SseB and various mutant forms of SseB was indicated by asterisks. The quantification of the signal intensities is shown in Additional file 1.

We observed that all deletion alleles led to the synthesis of corresponding proteins by a *Salmonella sseB *strain harboring the respective plasmids if grown under *in vitro *conditions inducing the expression of the SPI2 genes and the secretion of substrate proteins by the SPI2-T3SS (Fig. 2; see also Additional file 1). However, Western blot analyses indicated that the amounts of cell-associated SseB differed for the various deletion constructs. We also determined the proportion of SseB in the detached fraction, corresponding to secreted protein bound to surface appendages of *Salmonella*, and in the supernatant fraction corresponding to secreted proteins without association to the cell surface (Fig. 2). For *Salmonella *WT, a large proportion of secreted SseB was found in the detached fraction. No signal for SseB was observed for the *sseB *strain. An *sseB *strain complemented with p*sseB *showed an equal distribution of secreted SseB in the detached and supernatant fraction and the distribution observed for this strain would be relevant for comparison to *sseB *strains harboring plasmids for the expression of various deletion constructs. We observed that SseBΔ4, SseBΔ5 and SseBΔ6 were not secreted or only present in secreted fractions in minute amounts. In addition, the amounts of SseBΔ5 and SseBΔ6 were highly decreased in comparison to the WT or the complemented strains and signals in Western blots were only detected after extended exposure times. SseBΔ1 was only detected in the detached fraction but not the secreted fraction. The situation was opposite for SseBΔ2, which was only present in the supernatant fraction but not in the detached fraction. Control Western blots for DnaK indicated that low amounts of this cytoplasmic protein were present in the detached fraction, thus the amounts of SseB in the detached fraction of the SseBΔ1 are likely to be the result of cell lysis. The secretion and partitioning of SseBΔ3 and SseBΔC1 was similar to that of WT SseB. For SseBΔ7 and SseBΔN1, highly reduced secretion was observed and the larger proportion of the secreted protein was present in the supernatant fraction. These analyses indicate that synthesis and secretion was affected to a different extend by deletions and that secretion similar to WT is possible even with deletions of larger portions of the protein. The deletion of the coiled-coil domain had little effect on secretion and partitioning (SseBΔ3), while mutations affecting the putative transmembrane region abolished the secretion of the mutant variants of SseB (SseBΔ2 and SseBΔ4). An SseB variant that lacked the postulated chaperone binding site in the C-terminal region of SseB was still synthesized, but the amounts of this protein in the secreted fractions were highly reduced (SseBΔ7).

**Figure 3:**
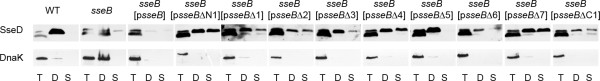
**Effect of various deletions in *sseB *on secretion and partitioning of SseD *in vitro***. *S*. Typhimurium WT or Δ*sseB *without plasmid, harboring plasmid p*sseB *for complementation of the *sseB *deletion, or plasmids for the expression of various *sseB *mutant alleles (p*sseB*Δx) were grown and analyzed as described for **Fig. 2**, but SseD was detected using a polyclonal antiserum raised against recombinant SseD. For the cytosolic portion of SseD, we observed a lower molecular weight form in addition to the protein found in the secreted fraction. The quantification of the signal intensities is shown in Additional file 1.

We then investigated the effect of the various deletions in SseB on the secretion of SseC and SseD and the partitioning of secreted SseC and SseD between the soluble and cell-bound fractions. For unknown reasons, larger amounts of DnaK were observed in the detached fraction of the *sseB *strain, but the mutation *per se *did not affect cell integrity since the complemented strains did not show increased release of cytosolic protein. In accordance with our previous report [7] we observed that the majority of SseD secreted by WT *Salmonella *is present in the detached fraction (Fig. 3). Strains expressing *sseB*Δ5, *sseB*Δ6 and, to a certain extend *sseB*Δ3 resulted in reduced amounts of the secreted SseD in the supernatant fraction. The expression of the other deletion alleles of *sseB *resulted in the presence of secreted SseD in the culture supernatant as well as in the detached fraction (Fig. 3). Deletions in *sseB*Δ5, *sseB*Δ6 affect the binding site for SseA that acts as chaperone for SseB and SseD [9]. The altered partitioning observed for strains expressing these alleles may be due to the altered binding of chaperone SseA to its targets and altered stability of these proteins. The partitioning of SseD in the complemented *sseB *strain was different from that observed for the WT strain and most of SseD was present in the total cell fraction rather than in the detached fraction. This may be due to the over expression of *sseB *in the *sseB *[p*sseB*] strain leading to more secretion of SseB in the supernatant and reduces the binding of SseB to the surface (compare Fig. 2). Therefore, the binding of SseD to the surface would be reduced and the release of SseD in the supernatant is increased. Most of the mutant alleles of *sseB *also resulted in higher amounts secreted SseD in the supernatant.

Similar effects were observed for the secretion and partitioning of SseC in strains expressing various alleles of *sseB *(data not shown).

### Effect of deletion of SseB domains on formation of translocon structures on Salmonella

**Figure 4:**
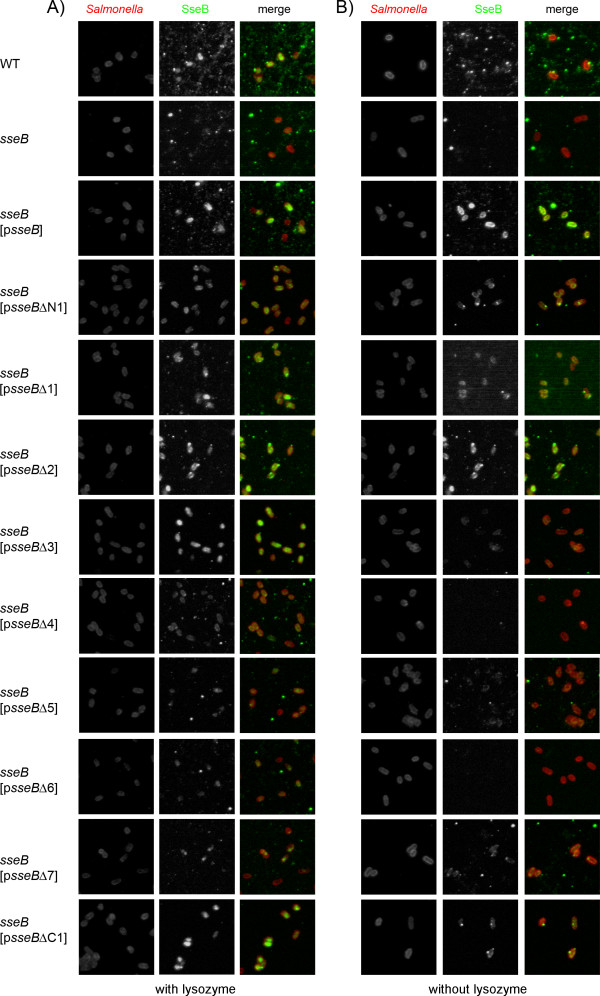
**Surface location of SseB variants under *in vitro *culture conditions**. *S*. Typhimurium wild type (WT), the *sseB *strain, or the *sseB *strain harboring p*sseB *for expression of WT *sseB*, or plasmids for the expression of various mutant alleles of *sseB *(p*sseB*Δx) were grown *in vitro *under conditions that induced the synthesis and secretion of SseB. At 8 h of culture, the bacteria were fixed on chitosan-pretreated cover slips. The bacterial cells were stained with rabbit anti-*Salmonella *O-1,4,5,12,27 antiserum conjugated with DyLight 547 NHS ester (red). SseB was immuno-stained using rabbit polyclonal antibody against recombinant SseB as primary antibody and anti rabbit Alexa488 was used as secondary antibody (green). A) Presence of SseB within the bacterial cytoplasm was investigated by immunofluorescence labeling of SseB after lysozyme permeabilization of the bacteria. B) For analyses of SseB secretion and surface location, the lysozyme treatment was omitted.

We have previously observed that secreted translocon proteins SseB, SseC and SseD were predominantly located in surface structures that occurred in single or low copy number resulting in a punctuated staining in immune-fluorescence analyses [7,8]. To test the effect of deletions in SseB on the formation of such surface structures, we used immunofluorescence to analyze various strains grown under secretion-inducing culture conditions (Fig. 4). The treatment of cells with lysozyme prior to immuno-labeling allowed the estimation of the cytoplasmic pool of SseB variants (Fig. 4A). The staining intensities for SseB observed correlated well with the data shown in Fig. 2. The investigation of surface-located SseB (Fig. 4B) indicated that WT SseB, SseBΔN1, SseBΔ1 and SseBΔC1 showed punctuate staining of single or low numbers of complexes per cell. A more intense and evenly distributed staining was observed for the p*sseB *complemented *sseB *strain and a strains expressing *sseB*Δ*2*. No or only very rare staining for SseB was found for the other mutant forms of SseB.

**Figure 5:**
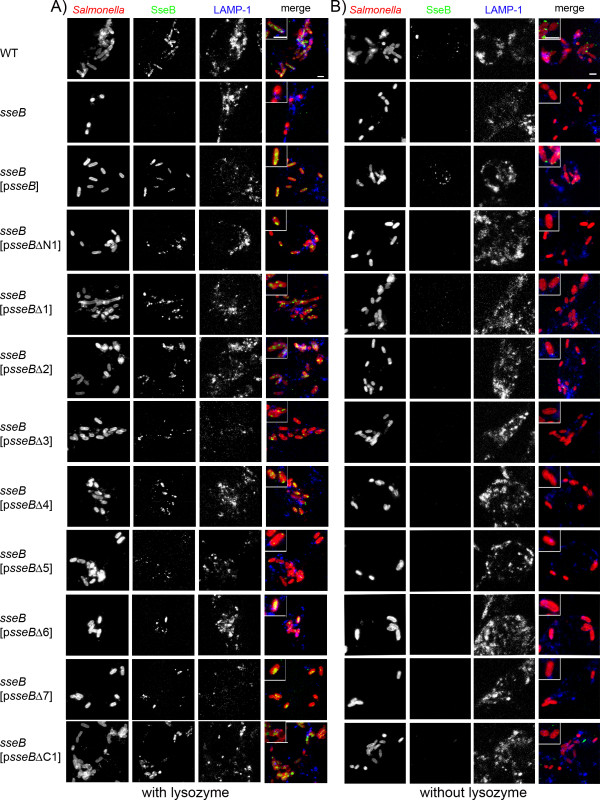
**Synthesis secretion and translocon formation of SseB variants by intracellular *Salmonella *after infection of RAW macrophages**. Macrophages were infected at a MOI of 25 with *S*. Typhimurium wild type (WT), the *sseB *strain, or the *sseB *strain harboring p*sseB *for expression of WT *sseB *or plasmids for the expression of various mutant alleles of *sseB *(p*sseB*Δx). At 6 h after infection, the infected cells were fixed with PBS containing 4% sucrose and 4% PFA and solubilized with 0.1% Triton X-100. SseB was immuno-stained using rabbit polyclonal antibody against recombinant SseB as primary antibody and anti rabbit Alexa488 was used as secondary antibody (green). *S*. Typhimurium was stained with rabbit anti-*Salmonella *O-1,4,5,12,27 antiserum conjugated with Dylight 547 NHS ester (red). To control the intracellular localization of the bacteria, the late endosomal/lysosomal membrane marker LAMP-1 was immuno-stained using monoclonal antibody and Cy5-conjugated secondary antibody (blue). A) Presence of SseB within the bacterial cytoplasm was investigated by immunofluorescence labeling of SseB after lysozyme permeabilization of intracellular bacteria. B) For analyses of SseB secretion and translocon formation lysozyme treatment was omitted. Note the labeling of SseB in the bacterial cytoplasm for all strain except for the *sseB *strain in A) and the absence or rare occurrence of punctuated surface labeling for all strains except WT and *sseB *[p*sseB*] in B).

We next investigated the function of the various mutant forms of SseB in intracellular *Salmonella *(Fig. 5). The analyses of the synthesis of SseB variants were performed by immuno-staining of intracellular *Salmonella *after lysozyme treatment in order to allow antibody to access the non-secreted pool of SseB in the bacterial cytoplasm (Fig. 5A). SseB staining was observed in the cytoplasm of the WT strain and absent for the *sseB *strain. For *sseB *strains harboring plasmids for the expression of WT *sseB *or any of the mutant alleles of *sseB*, signals in the bacteria were detected. However, the intensity of staining was different and rather weak labeling was observed for SseBΔ3, SseBΔ4 and SseBΔ5. Interestingly, in contrast to WT SseB that shows a homogenous distribution in the bacterial cytoplasm, we observed that SseB variants with deletions appeared to be concentrated at the poles of the bacterial cells (for example SseBΔ1 and SseBΔN1, Fig. 5A). Previous work showed that SseB can be detected by immuno-gold labeling on the surface of intracellular *Salmonella *and that SseB-positive proteinaceous structures correlated with needle-like extensions that were detected in low copy number by electron microscopy [8]. The immuno-labeling of intracellular *Salmonella *was repeated but lysozyme treatment was omitted in order to specifically label the SseB-containing structures on the bacterial surface. Staining of intracellular *Salmonella *WT for SseB confirmed the presence of SseB-containing structures on the bacterial surface (Fig. 5B). Not all of the intracellular bacteria were positive for SseB and positive cells showed one or two punctuate signals. Signals for SseB were entirely absent for the *sseB *strain, but present in the *sseB *strain complemented with p*sseB*. SseB-containing surface structures were very rare or not detectable in any of the *sseB *strains harboring plasmids for the expression of mutant alleles of *sseB*. The observations suggest that although deletions of domains in SseB in part are compatible with secretion and binding to the bacterial surface *in vitro*, formation of SseB-containing surface structure on intracellular bacteria did neither tolerate the absence of any domain in SseB nor N- or C-terminal truncations.

### Deletional analyses of SseD

**Figure 6:**
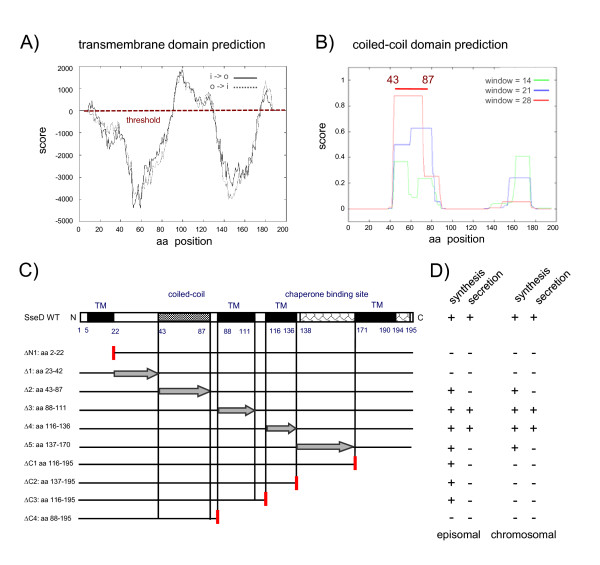
**Functional dissection of the putative translocon protein SseD**. Predictions of transmembrane domains (A) and coiled-coil regions (B) in SseD were performed as described for **Fig. 1**. Four transmembrane regions and one coiled-coil region were predicted for SseD using TMpred and COILS. The chaperone binding site for SseA is located within the C-terminus of SseD [10]. C) The location of TM and coiled-coil regions in wild-type SseD is indicated and the positions of internal deletions are indicated by arrows. N- or C-terminal truncations are indicated by vertical red lines. Plasmid-borne mutant alleles were also integrated into the chromosome applying λ. Red recombineering recombined with positive selection [29]. D) Analyses of synthesis and secretion of SseD variants under *in vitro *conditions. For the *in vitro *studies, bacteria harboring wild-type SseD, chromosomal or plasmid-borne deletion variants of SseD were analyzed as described in **Fig. 2**. Secreted proteins were detached from the cell surface or directly recovered from the supernatant by precipitation with TCA and subjected to Western blot analyses using antiserum raised against SseD. The presents or absence of SseD in the bacterial lysate or secreted fractions (detached fraction or supernatant) is indicated as + or -. The analyses of synthesis and secretion of plasmid-encoded variants of SseD are shown in Additional file 2.

We applied a similar deletion strategy to SseD. Based on the predictions of transmembrane regions (Fig. 6A; see also Additional file 2) and coiled-coil domains (Fig. 6B), variants of SseD were generated that lacked hydrophobic, putative TM domains, the coiled-coil domain, the chaperone-binding site or the N- or C-terminal parts of the protein (Fig. 6C). In addition to episomal expression of mutated *sseD*, exchange of the WT allele of *sseD *in the chromosome of *Salmonella *against mutant alleles was performed. The synthesis of SseDΔC1, SseDΔC2 and SseDΔC3 was observed if expressed by episomal genes, but not in strains with chromosomal deletions, likely due to lower expression levels. Synthesis of SseDΔN1, SseDΔ1 and SseDCΔ4 was not detectable at all. We observed that the larger number of the deletion constructs was not secreted under *in vitro *conditions (Fig. 6D, Suppl. Fig. 1). Secretion was only detected for the constructs SseDΔ3 and SseDΔ4 that lacked hydrophobic domains in the central region of the protein. The presence of the mutant alleles on episomal elements or in the chromosome had no effect on the efficiency of secretion. We have not been able to detect surface structures containing SseD for WT or mutant strains using the antiserum against SseD (data not shown). These observations show that the integrity of the primary sequence of SseD is of critical importance for the secretion of the protein and more sensitive to alterations compared to SseB.

### Effect of deletions of domains in SseB or SseD on translocation of a SPI2-T3SS effector protein

**Figure 7:**
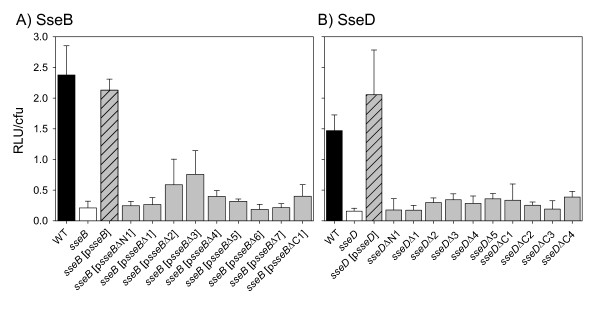
**Effect of mutations in SseB or SseD on translocation of the SPI2 effector protein SseJ**. Macrophages were infected at a MOI of 10 with *S*. Typhimurium wild type (WT), *sseB*, *sseB *[p*sseB*] or *sseB *harboring plasmids for expression of various *sseB *mutant alleles (*sseB *[p*sseB*Δx]) (A), or WT, *sseD*, *sseD *[p*sseD*], or various strains harboring chromosomal deletion in *sseD *(B). All strains harbored a chromosomal translational fusion of the firefly luciferase to codon 200 of *sseJ*. At 8 h (B) or 14 h (A) post infection, the host cells were lysed and the numbers of intracellular bacteria were determined. The rest of the cell lysates were centrifuged and the luciferase activity (relative light units = RLU) was measured in the supernatant in order to quantify the translocation of SseJ-Luc. The RLU per bacterium were calculated to compensate different replication rates of WT and the *sseB *mutant strains. Means and standard deviations of triplicate assays are shown and all experiments were performed at least twice.

We tested the ability of *Salmonella *strains expressing WT or various deletion variants of SseB (Fig. 7A) or SseD (Fig. 7B) to translocate a representative substrate protein of the SPI2-T3SS. The use of an SseJ-Luc fusion protein has previously described for the quantification of the amounts of translocated effector protein. Here, the amount of translocated SseJ-Luc was determined by measurements of luciferase activities in lysates of infected cells. As expected from previous studies on the role of SseB in translocation, Luc activities in the background of the *sseB *strain were highly reduced, while reporter activities for the *sseB *strain complemented with p*sseB *are similar to the levels for the WT strain. If the *sseB *strain was complemented with any of the deletion alleles of *sseB*, highly reduced levels of reporter activity are observed in host cell lysates. For most strains, the reporter activities were indistinguishable from those of the *sseB *mutant strain. Only the Luc activities for strains expressing *sseB*Δ2 and *sseB*Δ3 are slightly higher and reached about 20% of the activities of the WT strain.

For SseD, we observed that all deletions resulted in a reduction of the amount of translocated effector protein comparable to levels of the *sseD *strain. None of the strains harboring chromosomal deletions within *sseD *resulted in Luc activities higher than those of the *sseD *strain (Fig. 7B).

### Effect of deletions of domains in SseB or SseD on intracellular replication

**Figure 8:**
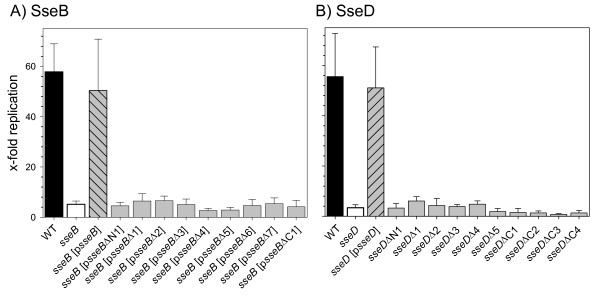
**Effect of mutations in SseB or SseD on intracellular replication of *Salmonella***. Macrophages were infected at a MOI of 1 with *S*. Typhimurium wild type (WT), *sseB*, *sseB *[p*sseB*] or *sseB *harboring plasmids for expression of various *sseB *mutant alleles (*sseB *[p*sseB*Δx]) (A), or WT, *sseD*, *sseD *[p*sseD*], or various strains harboring chromosomal deletion in *sseD *(B). Extracellular bacteria were killed by gentamicin treatment during 1 h post infection. Intracellular bacteria were quantified after host cell lysis with Triton X-100 at 2 h and 16 h post infection. The x-fold replication is the ratio of viable intracellular bacteria recovered at 16 h versus 2 h post infection. The replication rate was assessed in triplicates and the standard deviation of the mean was calculated. Means and standard deviations of triplicate assays are shown and all experiments were performed at least twice.

The effect of deletions in SseB or SseD on the intracellular replication in macrophages was quantified (Fig. 8). The intracellular replication of WT *Salmonella *and the complemented *sseB *strain was about 50- to 55-fold over a period of 14 h. The replication of the *sseB *strain without plasmid or with plasmids for the expression of any of the deletions alleles of *sseB *was reduced to an about 5-fold increase of the intracellular bacteria and no significant difference between the various constructs was observed (Fig. 8A). Similar characteristics were observed for strains expressing deletion alleles of *sseD *and none of the mutant strains showed intracellular replication that was above the level of the *sseD *strain (Fig. 8B).

These data indicate that SseB and SseD do not tolerate major alterations of the primary structure in order to fulfill their function as parts of the translocon of the SPI2-T3SS. The data also demonstrate that a fully functional translocon is required for the efficient intracellular replication. The residual ability of strains expressing *sseB*Δ2 or *sseB*Δ3 to translocate effector proteins appears to be insufficient to confer the ability of intracellular replication.

## Discussion

In this study we performed a structure-based functional dissection of the SPI2-T3SS translocon proteins SseB and SseD. Protein domains predicted as putative transmembrane regions or coiled-coil regions were deleted, as well as N- or C-terminal portions, and previously defined binding regions for the specific chaperone SseA [9,10].

The deletional and functional analyses described here clearly demonstrate the sensitivity of SseB and SseD against structural alterations. Many of the deletion variants lost the ability to be secreted by the SPI2-T3SS. However, we also identified a subset of deletion variants that were synthesized in quantities similar to the WT proteins, secreted under *in vitro *conditions and bound to the bacterial surface. The lack of the chaperone binding site in SseB led to reduced amounts of protein. We found that some mutant forms of SseB were on surface structures on bacteria grown *in vitro *(Fig. 4B), but not on intracellular *Salmonella *(Fig. 5B). Surface straining on intracellular bacteria was also absent for mutant forms of SseD. Although low levels of translocation of effector SseJ were possible in the presence of SseBΔ2 (deletion of transmembrane domain) or SseBΔ3 (deletion of coiled-coil domain), the corresponding strains was as highly attenuated in intracellular replication as the *sseB *mutant strain. This observation may indicate that the temporally and spatially coordinated translocation of several effector proteins is required for proper intracellular proliferation. The various mutant forms of SseD were neither assembled into polar organelles on the surface of intracellular bacteria, nor functional in translocation of effector proteins or in supporting the intracellular replication of *Salmonella *in macrophages.

A current model for the assembly of the translocon proposes the formation of a hetero-oligomeric platform at the tip of the T3SS filament [6,11]. The subunits LcrV (*Yersinia *spp.) or IpaD (*Shigella *spp.) assemble such platforms and based on sequence similarity, EspA of EPEC and SseB of the SPI2-T3SS are proposed to fulfill a similar function. LcrV, IpaD, SseB and EspA all harbor coiled-coil regions. The coiled-coil domain of EspA is essential for the assembly of the T3SS on the surface of EPEC [12]. In addition to function as a structural component of the translocon, EspA forms helical filaments [13], whereas a direct contribution of SseB to filament formation has not been observed. EspA filaments are thought to be optimized for the penetration of the mucus layer of the epithelium in order to establish contact with enterocytes for the translocation of effector proteins [13]. In contrast, the translocon of the SPI2-T3SS is assembled on bacteria within the SCV where no barrier might interfere with the insertion of the translocator pore into the target cell membrane. It was shown that SseB is present after secretion in a sheath-like structure on filamentous structures formed by the SPI2-T3SS *in vitro *[8]. Based on sequence similarity and previous functional characterization, SseC and SseD are likely to assemble the translocation pore of the SPI2-T3SS. We were not able to detect SseC on intracellular bacteria in the background of the various SseB deletion variants. In contrast, a defined punctuated staining for SseC was observed for WT and complemented *sseB *strain (data not shown). This indicates that mutations in SseB affect the organization of at least SseC on the surface of intracellular *Salmonella*. Further analysis of the tip of the SPI2-T3SS will require structural data for individual translocon proteins as well as for the oligomeric assembly of subunits SseB, SseC and SseD. Yet, the highly hydrophobic nature of SseC will impose serious limitations to biochemical approaches.

A functional dissection similar to our approach was performed by Chiu and Syu [14] for EspB from EHEC, the putative homologue of SseD. Although internal deletions and C-terminal truncations of similar extend to our deletions in SseD were generated, the effect of the mutations of EspB was far less dramatic and most mutant forms remained at least partial functions. To control if the loss of function phenotypes of *sseD *deletions were caused by the increased gene dosage due to episomal expression, deletion alleles were are also integrated in the native chromosomal context. However, SseD variants encoded by chromosomal alleles were also defective in the assembly of a functional translocation pore.

We propose that the function of the SPI2-T3SS of intracellular bacteria is more sensitive to structural alteration than the homologous components of T3SS of extracellular bacteria. Previous work revealed that only single or few copies of the T3SS exist and we assume that only these apparatuses mediated translocation [8]. In contrast, the T3SS systems of extracellular bacteria such as the EPEC LEE-T3SS, *Salmonella *SPI1-T3SS or *Shigella *Mxi/Spa-T3SS exist in multiple copies [15-17]. If mutations result in a reduced function of the translocon, this may be compensated by the large number of active T3SS.

Further characterization of translocation pores inserted into target cell membranes could also involve the analyses of protein interaction by pull down experiments, as previous applied to EPEC EspB and EspD interaction using GST tags [18]. We observed that translocon proteins of the SPI2-T3SS did tolerate the C-terminal addition of HA-tag, but not of Strep-tag or larger tags, thereby restricting the analysis of protein interaction (data not shown).

Interestingly, translocon proteins involved in bacterial invasion exhibit several functions in addition to effector translocation, e.g. binding to caspase-1 (IpaB, SipB) [reviewed in [19]] or actin binding (SipC) [20]. A contribution to the adhesion to host cells has also been observed for translocon subunits of the EPEC T3SS [21] and the SPI1-T3SS of *Salmonella *[22]. So far, no additional functions have been assigned to the SPI2 translocon protein SseB, SseC, SseD. The role of these proteins appears to be restricted to the basal translocon function.

The *Shigella *translocon protein IpaC requires polar localization in the bacterial cytoplasm for its secretion during the invasion process [23]. We observed that WT SseB was distributed homogeneously in the cytoplasm of intracellular *Salmonella*. Additional staining at various time points after infection of macrophages did not indicate a polar distribution of non-secreted SseB and SseC in the bacterial cytoplasm (data not shown). Polarized localization within intracellular bacteria was only observed for SseB deletion variants with defective functions. These observations suggest that the features of translocon proteins involved in invasion are distinct from those required for intracellular activities. *Shigella *and invasive *Salmonella *possess a large number of T3SS copies distributed all over the bacterial surface [15,16], but show a polar localization of the translocation complex [23,24]. In contrast, intracellular bacteria possess only one or few copies of the T3SS, but homogenous intracellular distribution of the translocon subunits [8]. The distribution of SseB may result from accumulation of redundant copies of SseB not required for translocon formation or may indicated a potential regulatory function on the expression or stability of other translocon subunits or effectors. The exact molecular mechanism behind this phenomenon has to be elucidated by future work.

## Conclusion

Taken together, our functional dissection reveals that SPI2-T3SS proteins SseB and SseD require all the distinct protein domains we identified for its proper function in translocon formation. Future analyses of the important interface between an intracellular pathogen and its host cell will require the analyses of roles of individual amino acid residues in the interaction of subunits and function of translocon subunits in mediating translocation of effector proteins.

## Methods

### Bacterial strains and growth conditions

*Salmonella enterica *serovar Typhimurium (*S*. Typhimurium) NCTC 12023 was used as wild type and mutant strains derived from *S*. Typhimurium 12023 are listed in Table 1. For standard cultivation, strains were grown in 3 ml Luria-Bertani (LB) medium in a roller drum (TC-7, New Brunswick) at 37°C. For the induction of expression of SPI2 genes and to trigger secretion by the SPI2-T3SS, minimal PCN-P media harboring phosphate starvation conditions at pH 5.8 was used. The minimal media contains 80 mM morpholineethanesulfonic acid (MES), 4 mM Tricine, 100 μM FeCl_3_, 376 μM K_2_SO_4_, 50 mM NaCl, 360 μM K_2_HPO_4_/KH_2_PO_4 _(pH 5.8), 0.4% glucose, 15 mM NH_4_Cl, 10 × micronutrients, 1 mM MgSO_4_, 10 μM CaCl_2 _and has been described in detail before [25]. For pre-culture PCN+P (25 mM phosphate) medium at pH 7.4, MES was replaced by morpholinepropanesulfonic acid (MOPS). If required, antibiotics carbenicillin or kanamycin were added to the various media at a concentration of 50 μg × ml^-1^.

**Table 1:** *Salmonella *strains used in this study

Designation	relevant characteristics	Reference
NCTC 12023	wild type	lab collection
MvP613	*sseJ*_200_::*luc aph*	Gerlach *et al*. [27]
MvP643	Δ*sseB*::FRT	Red deletion, this study
MvP723	Δ*sseB*::FRT *sseJ*_200_::*luc aph*	P22 of MvP613, this study
MvP1129	Δ*sseD*::FRT	Red deletion, this study
MvP1125	Δ*sseD*::FRT *sseJ*_200_::*luc aph*	P22 of MvP613, this study
MvP918	Δ*sseD*::*tetAR*	Red deletion, this study
MvP1048	Δ*sseD*_2-22*_	Red deletion, this study
MvP1049	Δ*sseD*_23-42_	Red deletion, this study
MvP1050	Δ*sseD*_43-87_	Red deletion, this study
MvP1051	Δ*sseD*_88-111_	Red deletion, this study
MvP1052	Δ*sseD*_116-136_	Red deletion, this study
MvP1053	Δ*sseD*_137-170_	Red deletion, this study
MvP1054	Δ*sseD*_171-195_	Red deletion, this study
MvP1055	Δ*sseD*_137-195_	Red deletion, this study
MvP1056	Δ*sseD*_116-195_	Red deletion, this study
MvP1057	Δ*sseD*_88-196_	Red deletion, this study
MvP1076	Δ*sseD*_2-22 _*sseJ*_200_::*luc aph*	P22 of MvP613, this study
MvP1078	Δ*sseD*_23-42 _*sseJ*_200_::*luc aph*	P22 of MvP613, this study
MvP1080	Δ*sseD*_43-87_*sseJ*_200_::*luc aph*	P22 of MvP613, this study
MvP1082	Δ*sseD*_88-111 _*sseJ*_200_::*luc aph*	P22 of MvP613, this study
MvP1084	Δ*sseD*_116-136 _*sseJ*_200_::*luc aph*	P22 of MvP613, this study
MvP1086	Δ*sseD*_137-170 _*sseJ*_200_::*luc aph*	P22 of MvP613, this study
MvP1088	Δ*sseD*_171-195 _*sseJ*_200_::*luc aph*	P22 of MvP613, this study
MvP1090	Δ*sseD*_137-195 _*sseJ*_200_::*luc aph*	P22 of MvP613, this study
MvP1092	Δ*sseD*_116-195 _*sseJ*_200_::*luc aph*	P22 of MvP613, this study
MvP1094	Δ*sseD*_88-196 _*sseJ*_200_::*luc aph*	P22 of MvP613, this study

* subscript denotes the first and last codon of the deletion

### Construction of mutant strains and plasmids

For the chromosomal deletion of the open reading frames of *sseB *or *sseD*, the 'one step inactivation' method of Datsenko and Wanner [26] was used. Following mutagenesis, the *aph *resistance cassette was removed by FLP-mediated recombination. The oligonucleotides used for mutagenesis are listed in Additional file 3.

For quantitative analyses of SPI2 effector translocation, the reporter fusion SseJ_200_-Luc [27] was transferred into the *sseB *(MvP643) or *sseD *(MvP1129) deletion mutant via P22 transduction according to standard methods [28]. Plasmids used in this study are listed in Table 2.

**Table 2:** Plasmids used in this study

Designation	relevant characteristics	Reference
pWSK29	low copy number vector	lab stock
pWSK30	low copy number vector	lab stock
p3232	pWSK30, *P*_*sseA *_*sseA sseB*	this study
p3320	Δ*sseB*_15-30 _*	p3232 derivative, this study
p3321	Δ*sseB*_38-57_	p3232 derivative, this study
p3322	Δ*sseB*_58-90_	p3232 derivative, this study
p3323	Δ*sseB*_38-90_	p3232 derivative, this study
p3324	Δ*sseB*_91-115_	p3232 derivative, this study
p3325	Δ*sseB*_116-136_	p3232 derivative, this study
p3326	Δ*sseB*_137-182_	p3232 derivative, this study
p3327	Δ*sseB*_2-14_	p3232 derivative, this study
p3328	Δ*sseB*_183-196_	p3232 derivative, this study
p3281	pWSK29, *P*_*sseA *_*sseD*	this study
p3329	Δ*sseD*_2-22_	p3281 derivative, this study
p3330	Δ*sseD*_23-42_	p3281 derivative, this study
p3331	Δ*sseD*_43-87_	p3281 derivative, this study
p3332	Δ*sseD*_88-111_	p3281 derivative, this study
p3333	Δ*sseD*_116-136_	p3281 derivative, this study
p3334	Δ*sseD*_137-170_	p3281 derivative, this study
p3335	Δ*sseD*_171-195_	p3281 derivative, this study
p3336	Δ*sseD*_137-195_	p3281 derivative, this study
p3337	Δ*sseD*_116-195_	p3281 derivative, this study
p3338	Δ*sseD*_88-195_	p3281 derivative, this study

* subscript denotes the first and last codon of the deletion

The plasmids for complementation of *sseB *and *sseD *were generated as follows: The wild-type sequence of *sseB *and the corresponding promoter region were amplified by PCR. The PCR product was purified using the Nucleotide removal kit (Qiagen), the purified DNA was digested by *Bam*HI/*Eco*RV and cloned into the *Bam*HI/*Eco*RV digested low-copy vector pWSK30. Cloning of *sseD *was performed similarly but the gene under the control of its own promoter was cloned via *Eco*RI/*Xba*I restriction sites into pWSK29. The generated plasmids were sequenced and introduced into the corresponding mutant strain by electroporation. The primers for cloning as well as sequencing are shown in Additional file 3.

Plasmid-borne deletion alleles of the *sseB *or *sseD *were generated by a PCR-based method using the QuikChange II XL Site-Directed Mutagenesis Kit according to the instruction of the supplier (# 200521-12, Stratagene, Heidelberg, Germany). All plasmids harboring mutant alleles were prescreened for successful mutagenesis, subsequently sequenced and introduced into the corresponding mutant strain by electroporation. Primers used for deletion, control PCR and DNA sequencing are listed in Additional file 3.

In order to move plasmid-borne *sseD *deletion alleles into the *Salmonella *chromosome, the λ Red system was applied in combination with positive selection for the loss of a tetracycline resistance cassette on Bochner-Maloy plates as described previously [29]. For amplification of the mutations affecting the inner region of *sseD*, the primer pair sseD-Del-Chrom-For and seq-rev were used. Fragments for deletions in the 5' or 3' region were amplified using sseD-delN1-chrom-For in combination with seq-rev or sseD-Del-Chrom-For together with sseD-del-C1 (2/3/4)-chrom-rev. All constructs were confirmed by sequencing. Sequences of primers used for deletion and sequencing are described in Additional file 3.

### Bioinformatics

For bioinformatic predictions in terms of coiled-coil domains and transmembrane regions of the SPI2 translocon proteins SseB and SseD, the freely available service of the Swiss EMBnet node server http://www.ch.embnet.org:http://www.ch.embnet.org/software/COILS_form.html, http://www.ch.embnet.org/software/TMPRED_form.html was engaged. The sequence manipulation suite of the Bioinformatic Organisation http://www.bioinformatics.org/sms/prot_mw.html was conducted in order to calculate the molecular weight of the SseB and SseD wild-type proteins as well as of the mutant variants of both proteins.

### Analyses of in vitro protein expression, surface attachment and secretion

For the *in vitro *analyses of the expression, surface-attachment and secretion of SseB and SseD as well as the plasmid-borne or chromosomal derived mutant variants, the secretion assay described by Nikolaus *et al*. [7] was modified. *Salmonella *strains were pre-cultured overnight in PCN+P (25 mM P_i_) pH 7.4, diluted 1:50 in 400 ml PCN-P media at pH 5.8 and incubated 7 h in a shaker platform with agitation at 150 rpm at 37°C. For analyses of protein synthesis, aliquots of 1 ml bacterial culture were pelleted by centrifugation in a table top centrifuge (Sigma 1-13) for 15 min at max. speed. The pellets were resuspended in sample buffer (12.5% glycerol, 4% SDS, 50 mM Tris-HCl pH 6.8, 2% β-mercaptoethanol, 0.01% bromophenol blue) according to the optical density (OD_600 _of 1 ml of culture × 100 = × μl of sample buffer) and heated at 95°C for 5 min. The remaining bacterial cultures were pelleted in a Sorvall RC5C centrifuge (ThermoFisher Scientific) at 6,000 × g for 30 min at 4°C. For extraction of secreted proteins, the supernatant was passed through a 0.2 μm Zap-cup sterile filter (10443401 Whatman Schleicher&Schuell) and proteins were precipitated with trichloroacetic acid (TCA, 10% [wt/vol] final concentration) over night at 4°C. The pellet was resuspended in 20 ml PBS in a 50 ml centrifuge tube (Falcon, BD) and vigorously mixed on a Vortex mixer (Vortex Genie 2, Scientific Industries) for 60 s at full speed in order to recover cell surface attached proteins (detached fraction). Bacteria were harvested by centrifugation at 8,000 × g 30 min at 4°C. Residual bacteria were removed by passing the supernatant through a 0.2 μm filter (Corning) and proteins were precipitated with 10% [wt/vol] TCA over night at 4°C. The TCA precipitates of the supernatant and the detached fraction were pelleted by centrifugation for 45 min at 10,000 × g at 4°C. The pellet was washed twice with ice-cold acetone and recovered by centrifugation for 30 min at 10,000 × g at 4°C. The final pellet was air dried, resuspended in × μl sample buffer corresponding to the volume of the pellet and heated at 95°C for 5 min.

Expression, surface-attachment and secretion protein profiles of wild-type SseB or SseD and mutant variants, were analyzed by SDS-Page using Tris-Tricine gels (12%) according to the method of Schägger and von Jagow [30]. For Western blotting, the semi-dry blotting procedure described by Kyhse-Andersen [31] was performed with slight modifications. The proteins were transferred onto 0.2 μm nitrocellulose membranes (Schleicher & Schüll) in Towbin buffer according to standard protocols [32].

For detection of SseB and SseD on Western blots, purified polyclonal rabbit antisera were used [7]. Mouse anti DnaK (Biotrend, Cologne, Germany) antibody was used to control equal loading of bacterial lysates as well as release of cytosolic protein into the detached fraction and the culture supernatant due to bacterial cell lysis. As secondary antibodies, horseradish peroxidase-conjugated goat anti-rabbit IgG and goat anti-mouse IgG (HRP, Jackson) were used. The blots were incubated for 1 min with Pierce^® ^ECL Western Blotting Substrate (32209, ThermoScientific) and exposed to X-ray films (Hyperfilm, GE, Freiburg, Germany).

### Cell culture and infection procedure

For infection experiments, the murine monocyte cell line RAW264.7 was cultured in DMEM (E15-843, PAA, Pasching, Austria) supplemented with 10% FCS (Sigma-Aldrich) and 2 mM Glutamax (Invitrogen) at 37°C in 5% CO_2_and 90% humidity. The cells were used for experiments up to passage number 25. Cells were seeded in 24 well plates (Greiner bio-one) one day before infection and allowed to duplicate. Bacteria were grown overnight at 37°C and stored at 4°C until use. Cultures were adjusted to OD_600 _= 0.2 (equivalent to 4 × 10^8 ^bacteria × ml^-1^) in PBS and a master-mix of the bacteria in DMEM at a desired multiplicity of infection (MOI) was prepared directly prior infection. 300 μl bacteria suspension was added per well. Bacteria were centrifuged onto the macrophages for 5 min at 500 × g and phagocytosis of the bacteria were allowed for 25 min at 37°C. After infection, macrophages were washed two times with PBS and residual extracellular bacteria were killed by the addition of 100 μg ml^-1 ^gentamicin dissolved in DMEM for 1 h at 37°C. Subsequently, 15 μg × ml^-1^gentamicin in DMEM was added for the remaining infection period. Depending on the experiment, the infected cells were lysed or fixed various times points post infection as described below.

### Intracellular replication assay and quantitative analyses of SPI2 effector translocation

In order to assess intracellular replication, 2 × 10^5 ^macrophages were seeded and a MOI of 1 was used for infection. 2 h and 16 h post infection, the infected cells were washed twice with PBS and lysed with 500 μl of 0.1% Triton X-100 10 min at RT. The lysates were adjusted to 1 ml with PBS and serial dilutions were plated onto MH plates in order to determine the colony forming units (CFU) of viable bacteria. The x-fold intracellular replication was defined by calculating the ratios of CFU counts at 16 h and 2 h after infection.

Quantification of intracellular SPI2 effector translocation was carried out as described previously [27]. Briefly, about 8 × 10^5 ^macrophages were infected with various *Salmonella *strains all harboring a chromosomal SseJ_200_-luciferase reporter fusion protein at a MOI of 10. 8 h and 14 h post infection, respectively, lysis of infected cells was performed for 15 min with shaking at RT using 100 μl of eukaryotic lysis buffer (#1669893, Roche). 10 μl lysate was used for preparation of various dilution series in PBS that were plated onto MH plates in order to count intracellular cfu. The remaining lysate was centrifuged at maximal speed for 3 min in a table top centrifuge (1-13, Sigma). Triplicates of 25 μl supernatant were applied to 96 well microtiter plates (Microfluor, Dynatech) and 50 μl luciferase reagent was added directly before the measurement was started. Luciferase activity of translocated SseJ-Luc effector protein was measured using a TopCount instrument (PerkinElmer) and expressed as Relative Light Units (RLU). The RLU per intracellular bacterium was calculated to adapt differences in replication.

### Immunofluorescence analyses of intracellular SseB expression and secretion

For immuno-staining of SseB on the bacterial surface or within the bacterial cytosol after infection of macrophages the method of Schlumberger *et al*. [24] was applied. Briefly, macrophages were seeded on cover slips in 24 well plates at a density of 1 × 10^5 ^cells and infection was conducted at a MOI of 25. 6 h post infection, the medium was removed and the infected macrophages were fixed directly with 4% *para*-formaldehyde (PFA) and 4% sucrose in PBS for 20 min at RT. Subsequently, the cells were washed three times with PBS, stored overnight in 4% sucrose in PBS and incubated in 20% sucrose in PBS for 10 min at RT. Following three washing steps with PBS, the cells were permeabilized with buffer A (50 mM EDTA, 20 mM Tris-HCl, 1.8 g/l glucose, pH 8.0) containing freshly added 0.1% Triton X-100 for 5 min at RT. Buffer A was replaced by three washing steps with buffer B (10 mM EDTA, 25 mM Tris-HCl, 1.8 g/l glucose, pH 8.0) and buffer B plus 5 g/l lysozyme for staining of proteins in the bacterial cytosol or without lysozyme for staining of intracellular secreted proteins was added for 1 h at 4°C. Cells were washed again with PBS and incubated for 1 h at RT in blocking solution (10% goat serum, 1% bovine serum albumin, 4% sucrose in PBS). SseB was stained using polyclonal antisera against recombinant SseB from rabbit [7] and anti-rabbit Alexa488 (Molecular Probes, Invitrogen). *S*. Typhimurium was stained with rabbit anti-*Salmonella *O1,4,5,12,27 antiserum (Difco) conjugated with DyLight 547 NHS ester (Pierce). The lysosome-associated membrane protein 1 (LAMP-1) that is associated with SCV in infected cells was stained using a monoclonal antibody H4A3 from rat (1:100, developed by J.T. August, J.E.K. Hildreth, was obtained from the Developmental Studies Hybridoma Bank developed under the auspices of the NICHD and maintained by The University of Iowa, Department of Biology, Iowa City, IA 52242) and anti rat Cy5 (1:500, Jackson). All antibodies were diluted in blocking solution. Following immuno-staining, the coverslips were mounted on Fluoprep (bioMèrieux) and sealed with Entellan (Merck). Images of the samples were recorded using a confocal laser-scanning microscope (Leica TCS-NT).

## Authors' contributions

SUH and MH designed experiments, SUH performed experimental work, SUH and MH analyzed data and wrote the manuscript. All authors read and approved the final manuscript.

## Supplementary Material

Additional file 1 **Effect of various deletions in *sseD *on synthesis and secretion of SseD *in vitro***. *S*. Typhimurium WT or Δ*sseD *without plasmid, harboring plasmid p*sseD *for complementation of the *sseD *deletion, or plasmids for the expression of various *sseD *mutant alleles (p*sseD*Δx) were grown in 400 ml minimal medium PCN-P (0.4 mM) at pH 5.8 to induce SPI2 expression as well as protein secretion by the SPI2-T3SS. For analyses of protein synthesis, equal amounts of bacterial cells as adjusted by OD_600 _were harvested and resuspended in SDS-PAGE sample buffer (total cell fraction). Secreted protein bound to the bacterial surface was released by mechanical shearing and precipitated from bacteria-free supernatant (detached fraction) and secreted proteins in the supernatant were precipitated by addition of 10% TCA (final concentration). For Western blot analysis, samples corresponding to equal amount of bacteria or supernatant were separated by SDS-PAGE and transferred to nitrocellulose membranes and protein was detected with antiserum raised against SseD. As loading control and control for cell lysis, the bacterial heat shock protein DnaK was detected. In total cell lysates, we observed a non-specific binding (indicated by the asterisk). Click here for file

Additional file 2 **Quantification of the effects of various deletions in *sseB *on synthesis and secretion of SseB *in vitro *and on secretion and partitioning of SseD *in vitro***. The signals of Western blot shown in Fig. 2 for the secretion and partitioning of SseB and mutant variant and the Western blot shown in Fig. 3 for the effector of deletions in SseB on secretion an partitioning of SseD were quantified. Densitometry was performed using ImageJ software http://rsbweb.nih.gov/ij/ and signal intensities were normalized to the total cell fraction set to 100%. Click here for file

Additional file 3 **Oligonucleotides used in this study**. The designation and sequence of oligonucleotides used for mutagenesis, strain construction and sequencing is shown. Click here for file
