# Supplementary material for: Functional dissection of translocon proteins of the Salmonella Pathogenicity Island 2-encoded type III secretion system
Source: BMC Microbiol. 2010 Apr 8;10:104. doi: 10.1186/1471-2180-10-104 (PMC2873485; doi:10.1186/1471-2180-10-104)
Supplement: Additional file 1 — Effect of various deletions in sseD on synthesis and secretion of SseD in vitro. S. Typhimurium WT or ΔsseD without plasmid, harboring plasmid psseD for complementation of the sseD deletion, or plasmids for the expression of various sseD mutant alleles (psseDΔx) were grown in 400 ml minimal medium PCN-P (0.4 mM) at pH 5.8 to induce SPI2 expression as well as protein secretion by the SPI2-T3SS. For analyses of protein synthesis, equal amounts of bacterial cells as adjusted by OD600 were harvested and resuspended in SDS-PAGE sample buffer (total cell fraction). Secreted protein bound to the bacterial surface was released by mechanical shearing and precipitated from bacteria-free supernatant (detached fraction) and secreted proteins in the supernatant were precipitated by addition of 10% TCA (final concentration). For Western blot analysis, samples corresponding to equal amount of bacteria or supernatant were separated by SDS-PAGE and transferred to nitrocellulose membranes and protein was detected with antiserum raised against SseD. As loading control and control for cell lysis, the bacterial heat shock protein DnaK was detected. In total cell lysates, we observed a non-specific binding (indicated by the asterisk). [file 1471-2180-10-104-S1.DOC]

**Additional File 2: Quantification of the effects of various deletions in *sseB* on synthesis and secretion of SseB *in vitro* and on secretion and partitioning of SseD *in vitro.***

**Strain fraction^*^ SseB secretion^**^ SseD secretion^**^**

WT T 100.00 100.00

D 357.34 1651.34

S 40.47 0.00

*sseB* T 100.00 100.00

D 336.57 250.47

S 46.05 12.38

*sseB* [p*sseB*] T 100.00 100.00

D 102.06 0.86

S 61.31 0.43

*sseB*ΔN1 T 100.00 100.00

D 3.97 53.56

S 17.75 46.60

*sseB*Δ1 T 100.00 100.00

D 13.13 25.47

S 0.00 5.82

*sseB*Δ2 T 100.00 100.00

D 0.00 14.04

S 11.94 6.92

*sseB*Δ3 T 100.00 100.00

D 51.11 33.87

S 21.51 3.77

*sseB*Δ4 T 100.00 100.00

D 0.00 42.81

S 4.00 25.73

*sseB*Δ5 T 100.00 100.00

D 9.45 90.63

S 0.00 0.00

*sseB*Δ6 T 100.00 100.00

D 0.00 9.96

S 0.00 3.12

*sseB*Δ7 T 100.00 100.00

D 20.19 47.55

S 47.10 42.92

*sse*BΔC1 T 100.00 100.00

D 53.11 12.93

S 30.06 6.73

* T, total cell fraction; D, detached fraction; S, supernatant fraction

** percentage of the signal intensity quantified for fractions of the WT strain
