# Supplementary figures and images for: Functional dissection of translocon proteins of the Salmonella Pathogenicity Island 2-encoded type III secretion system
Source: BMC Microbiol. 2010 Apr 8;10:104. doi: 10.1186/1471-2180-10-104 (PMC2873485; doi:10.1186/1471-2180-10-104)

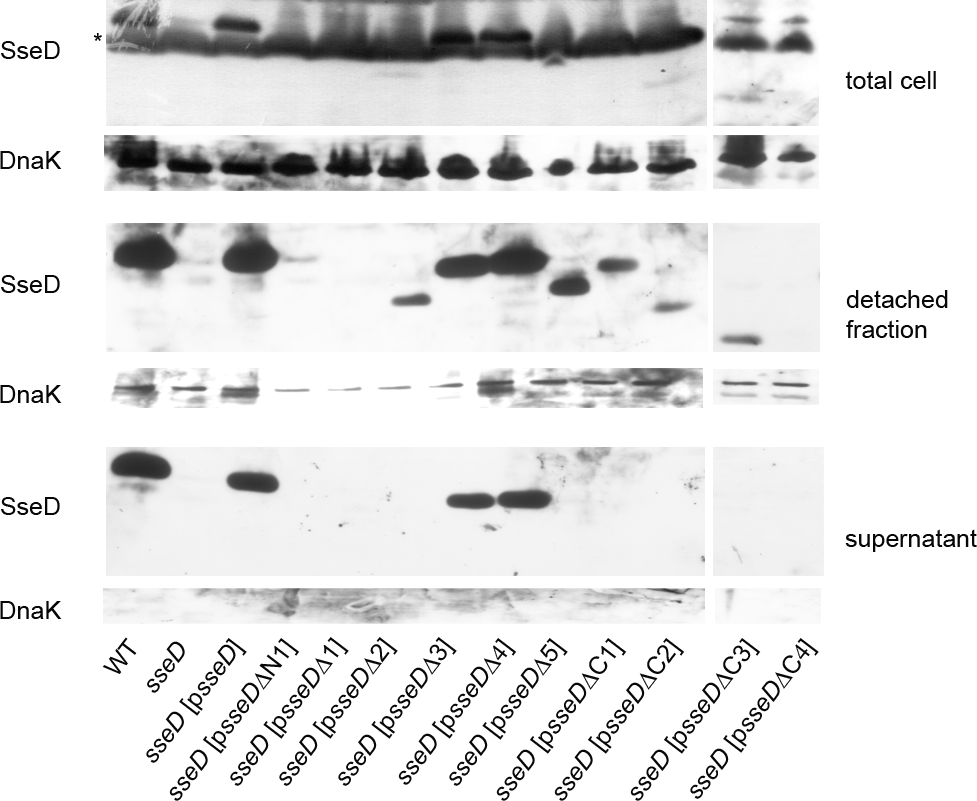

Supplement: Additional file 2 — Quantification of the effects of various deletions in sseB on synthesis and secretion of SseB in vitro and on secretion and partitioning of SseD in vitro. The signals of Western blot shown in Fig. 2 for the secretion and partitioning of SseB and mutant variant and the Western blot shown in Fig. 3 for the effector of deletions in SseB on secretion an partitioning of SseD were quantified. Densitometry was performed using ImageJ software http://rsbweb.nih.gov/ij/ and signal intensities were normalized to the total cell fraction set to 100%. [file 1471-2180-10-104-S2.TIFF]
