# Supplementary material for: Functional dissection of translocon proteins of the Salmonella Pathogenicity Island 2-encoded type III secretion system
Source: BMC Microbiol. 2010 Apr 8;10:104. doi: 10.1186/1471-2180-10-104 (PMC2873485; doi:10.1186/1471-2180-10-104)
Supplement: Additional file 3 — Oligonucleotides used in this study. The designation and sequence of oligonucleotides used for mutagenesis, strain construction and sequencing is shown. [file 1471-2180-10-104-S3.DOC]

#### Additional File 1: Oligonucleotides used in this study

#### Designation Sequence*

#### Deletion primers

#### SseB-Red-Del-For 5` GTGGAGATACCGTCAGGAAAAACAAAAAGGTAAAGCATAATGgtgtaggctggagctgcttc 3´

#### SseB-Red-Del-Rev 5` GCCTGTTGTAGGGTCGGGTCTTTTTTCATGAGTACGTTTTcatatgaatatcctccttag 3´

#### sseD-Red-Del-For 5` TTAATCTGAGGATAAAAATATGGAAGCGAGTAACGTAGCACtgtgtaggctggagctgctt 3´

#### SseD-RedDel-rev043 5` GTAACCATTGCTCTATTTCTTGCACCATGTTTACCTCGTTAAcatatgaatatcctccttag 3´

#### sseD-tetA/R-for 5` GAGAATAGCTGGCTATCGCGCTTAATCTGAGGATAAAAATctaagcacttgtctcctg 3´

#### sseD-tetA/R-rev 5` CACCCGACAACATTGCGCCACCCGCAGTAATCATTGATGCttaagacccactttcac 3´

#### Cloning primers

#### RT-ssaD-for 5` gacttattggtacgagaagtgcagg 3´

#### sseB EcoRV rev 5` ttt*gatatc*tcatgagtacgttttctgcgc 3´

#### SseD-for-EcoRI-2 5` gcg*gaattc*ggataaaaatatggaagcgag 3´

#### SseD-Rev2-XbaI 5` gca*tctaga*ttacctcgttaatgcccgg3´

#### del-sseB15-30-for 5` ggggaagtcaaaaccctattgatgacttatcccagcaaaatcc 3´

#### del-sseB15-30-rev 5` ggggaagtcaaaaccctattgatgacttatcccagcaaaatcc 3´

#### del-SseB38-57-for 5` gacttatcccagcaaaataataataaatttattgaagtccag 3´

#### del-SseB38-57-rev 5` ctggacttcaataaatttattattattttgctgggataagtc 3´

#### del-SseB58-90-for 5` ggttattcaggctatcgcaggggatgctaaaaccaaagag 3´

#### del-SseB58-90-rev 5` ctctttggttttagcatcccctgcgatagcctgaataacc 3´

#### del-SseB39-90-for 5` gacttatcccagcaaaatggggatgctaaaaccaaagag 3´

#### del-SseB38-90-rev 5` ctctttggttttagcatccccattttgctgggataagtc 3´

#### del-SseB91-115-for 5` gtgattgctaaagcagccaaaggtatgaccattgatgatta 3´

#### del-SseB91-115-rev 5` taatcatcaatggtcatacctttggctgctttagcaatcac 3´

#### del-SseB116-136-for 5` gataatggtattctcatcgatctacaggcgatcaaagcggct 3´

#### del-SseB116-136-rev 5` agccgctttgatcgcctgtagatcgatgagaataccattatc 3´

#### del-SseB137-182-for 5` gggaagctggataaaggtggctggggggaaatttccagtatg 3´

#### del-SseB137-182-rev 5` catactggaaatttccccccagccacctttatccagcttccc 3´

#### del-SseBΔN1(2-14)-for 5` caaaaaggtaaagcataatggtgtttaaaaatagcttcggc 3´

#### del-SseBΔN1(2-14)-rev 5` gccgaagctatttttaaacaccattatgctttacctttttg 3´

#### Designation Sequence*

#### del-SseBΔC1(183-196)-for 5` ctgacagggcttatcagtaagtgagatatcaagcttatcgat 3´

#### del-SseBΔC1(183-196)-rev 5` atcgataagcttgatatctcacttactgataagccctgtcag 3´

#### SseD-delN1-for 5` gaattcggataaaaatatgtctccctccggggagggaatgg 3´

#### SseD-delN1-rev 5` ccattccctccccggagggagacatatttttatccgaattc 3´

#### sseD-del1-for 5` caccttcttccactccaatgaagctaatggagcttgc 3´

#### sseD-del1-rev 5` gcaagctccattagcttcattggagtggaagaaggtg 3´

#### sseD-del2-for 5` gttatttgatgatatctgggcatcaatgattactgcggg 3´

#### sseD-del2-rev 5` cccgcagtaatcattgatgcccagatatcatcaaataac 3´

#### sseD-del3-for 5` caattgatgaagcgtttagagaaaccggtcttatagcgg 3´

#### sseD-del3-rev 5` ccgctataagaccggtttctctaaacgcttcatcaattg 3´

#### sseD-del4-for 5` ggcggggaaaccggtcttcaacgtcaaagtgatcaag 3´

#### sseD-del4-rev 5` cttgatcactttgacgttgaagaccggtttccccgcc 3´

#### sseD-del5-for 5` cctgggggctggtgtagcgattatgcagcaaatcatcg 3´

#### sseD-del5-rev 5` cgatgatttgctgcataatcgctacaccagcccccagg 3´

#### sseD-delC1-for 5` gaaattatggagaaagcaactgaataatctagagcggccgcc 3´

#### sseD-delC1-rev 5` ggcggccgctctagattattcagttgctttctccataatttc 3´

#### sseD-delC2-for 5` cctgggggctggtgtagcgtaatctagagcggccgcc 3´

#### sseD-delC2-rev 5` ggcggccgctctagattacgctacaccagcccccagg 3´

#### sseD-delC3-for 5` ggcggggaaaccggtctttaatctagagcggccgcc3´

#### sseD-delC3-rev 5` ggcggccgctctagattaaagaccggtttccccgcc 3´

#### sseD-delC4-for 5` caattgatgaagcgtttagataatctagagcggccgcc 3´

#### sseD-delC4-rev 5` ggcggccgctctagattatctaaacgcttcatcaattg 3´

#### sseD-Del-Chrom-For 5` agcgttcaggcgagaatagctggctatcgcgcttaatctgaggataaaaatatggaagcg 3´

#### sseD-delN1-chrom-For 5` gcgttcaggcgagaatagctggctatcgcgcttaatctgaggataaaaatatgtctccc 3´

#### sseD-del-C1-chrom-rev 5` tgatgccgacgtaaccattgctctatttcttgcaccatgtttattcagttgctttctcca 3´

#### sseD-delC2-chrom-rev 5` tgatgccgacgtaaccattgctctatttcttgcaccatgtttacgctacaccagccccc ´

#### sseD-delC3-chrom-rev 5` tgatgccgacgtaaccattgctctatttcttgcaccatgtttaaagaccggtttccccgc 3´

#### sseD-delC4-chrom-rev 5` tgatgccgacgtaaccattgctctatttcttgcaccatgtttatctaaacgcttcatcaa 3´

#### Designation Sequence*

#### Control and sequencing primers

#### RT-sseA-for 5` cgggctaaggtgagtcaacagc 3´

#### k1-red-del 5` cagtcatagccgaatagcct 3´

#### RT-sscA-rev 5` catcttttctgcacgctgtcg 3´

#### SseD-For-PstI 5` tgg*ctgcag*atggaagcgagtaacgtag 3´

#### TetAR-Red-Check-For 5` gatcaagagcatcaagtcgc 3´

#### sseB EcoRV rev 5` ttt*gatatc*tcatgagtacgttttctgcgc 3´

#### SseB-Seq-For 5` cttaatgctcaagccccggtgg 3´

#### T3-Seq 5` aattaaccctcactaaagg 3´

#### sseA-Xba1-FW 5` gtatctagatcgtgtatatggaggggaatg 3´

#### Seq-For 5` cgccagggttttcccagtcacgac 3´

#### Seq-Rev 5` agcggataacaatttcacacagga 3´

#### RT-sseC-for 5` ggctatcagatgtgcaaccgag 3´

#### RT-sseE-rev 5` gcctcaatacgatagatcaccagg 3´

#### sseB-seq-rev-chromosomal 5` gccgccacgccggaaaaacc 3´

#### sseD-seq-rev-chromosomal 5` atgcaggctcagtaaacacc 3´

#### sseD-seq-for 5` cactatagtagcgttcaggcg 3´

#### ^*^ Complementary sequences in deletion primers are in capital letters and introduced restriction sites are in italics.
